# The Impact of EFL Teachers’ Open-Mindedness and Immediacy on Their Social Intelligence: A Theoretical Review

**DOI:** 10.3389/fpsyg.2022.872250

**Published:** 2022-04-08

**Authors:** Zhan Cui

**Affiliations:** School of Foreign Languages, Xinyang Normal University, Xinyang, China

**Keywords:** EFL teachers, immediacy, positive psychology, open-mindedness, social intelligence, interpersonal communication skills

## Abstract

The role of psycho-emotional and social factors in determining teachers’ pedagogical success has been largely endorsed in the literature. This highlights the necessity of improving EFL teachers’ awareness and knowledge of various factors related to classroom interaction and interpersonal communication. Despite the existence of some L2 studies on interpersonal communication skills after the popularity of positive psychology, the interplay of variables that directly reflect classroom interactions and their linkage to one’s intelligence(s) has been overlooked, to date. To fill this gap, the present mini review presented the theoretical and empirical foundations of three significant teacher-related variables, namely open-mindedness, immediacy, and social intelligence. In so doing, their relevant definitions, conceptualizations, models, and correlates were provided. Finally, the study offered some practical implications and suggestions for EFL stakeholders and researchers who can improve their knowledge and use of social-based variables in second/foreign language education.

## Introduction

Definitely, in EFL contexts where the students mostly depend on teachers, teachers are seen as the most significant stakeholders who can considerably affect the rate and quality of classroom instruction and communication (Pishghadam et al., 2021). Therefore, in such classes with students holding dissimilar beliefs and values, the decisions that teachers make extremely influence academic achievements and perceptions (Danielson, 2002). Other than instructional features, teachers’ decisions, practices, behavioral, and psychological states are critical in L2 education (Burroughs et al., 2019). To survive and generate optimum outcomes in L2 education which is constantly dealing with numerous linguistic, psychological, emotional, and cultural factors, there must be established an academic milieu in which the diversity of beliefs and viewpoints of different stakeholders are cared about and respected. This necessitates teachers’ open-mindedness and its inculcation in their students. Open-mindedness is a characteristic that represents both the cognitive and civic excellence of an individual (Riggs, 2010). An open-minded EFL teacher is someone who is fine with new, unfamiliar, and different ideas and strategies. He/she looks at the issues from various angles respecting others’ opposing opinions (Nurfaidah, 2018). This feature requires EFL teachers to hold their own teaching beliefs lightly and consider a room for others’ perspectives and ideas by establishing a democratic context with diversity awareness and appreciation.

One of the upshots of open-minded L2 education is the creation of immediacy or positive rapport in the classroom among EFL teachers and students (Delos Reyes and Torio, 2020). The concept of teacher immediacy is described as a range of verbal and non-verbal behaviors and strategies that teachers employ to establish a sense of closeness and proximity with learners (Dickinson, 2017). It was first projected by Mehrabian (1969) as a communication behavior to explain the amount of closeness and rapport between people (Finn and Schrodt, 2012). Different studies in the pertinent literature have proved that teacher immediacy leads to learners’ empowerment (Cakir, 2015), classroom engagement (Marx et al., 2016; Derakhshan, 2021), sustained attention (Bolkan et al., 2017), improved clarity and credibility (Zheng, 2021), and diminishes anxiety (Kelly et al., 2015). Despite these studies, the effect of immediacy on EFL teachers, especially their psycho-emotional variables, has been limitedly (if any) explored. One such factor can be teachers’ social intelligence (SI) or their capability to act wisely in human interactions and communications (Thorndike, 1920). It is an interpersonal form of intelligence resembling emotional intelligence (EQ) and social competence that highlights the importance of knowing and respecting culturally different behaviors, cultures, sub-culture(s), diversity-awareness, and collaboration across countries (Albrecht, 2006).

As L2 educational systems worldwide are now following interaction-oriented syllabi that stress both teachers’ and students’ dialogical interactions, it is essential for educators to have high levels of interpersonal communication skills (e.g., clarity, credibility, immediacy) and SI that capitalize on interpersonal diversity and knowledge of the social conducts of interaction (Genç and Boynukara, 2017; Xie and Derakhshan, 2021). Although EFL classrooms are acknowledged as social settings requiring communication skills, running investigations on constructs related to this domain are regrettably scant. To bridge this lacuna, the current mini review aimed to present the theoretical and empirical underpinnings of three social and interaction-based variables in EFL contexts (open-mindedness, immediacy, and SI) and their interaction. It also provides some useful implications and directions for future research in this academic zone.

## Background

### The Definition of Open-Mindedness

The concept of open-mindedness, as opposed to closed-mindedness, refers to the way and degree of receptiveness and respect that a person has for new ideas and opinions (Tjosvold and Poon, 1998; Riggs, 2010). Likewise, Baehr (2011) described an open-minded individual as someone who typically goes beyond or momentarily sets aside his own personal attitudes to give an unbiased hearing to the rational conflict. This sense emanates from one’s awareness and acknowledgment of the natural weakness of his/her beliefs (Riggs, 2010). Hence, open-minded people are more prone to take novel and alternative viewpoints seriously and listen to them eagerly (Kwong, 2016).

### Open-Mindedness and Teaching

In second/foreign language education which is full of adversities and challenges (Li, 2021), the construct of open-mindedness is an essential factor to be cultivated in teachers and students. It is an indication of the cognitive and social growth of these stakeholders regarding the acceptance and tolerance of opposing views. As research proves, open-mindedness is a unique characteristic of teachers’ reflectivity and critical thinking (Nurfaidah, 2018; Sadeghi et al., 2020). In teaching, this personal attribute is significant in that teachers and students coming from diverse socio-cultural backgrounds may have different and, in some cases, conflicting viewpoints. Consequently, it is pivotal to learn how to face and cope with diversities in opinions and ideas and establish a positive, democratic classroom climate in which “difference does not mean wrong.” In classroom context, as a miniature of social world, students and teachers must raise their awareness and receptiveness to alternative and opposing viewpoints so that they can learn to live a harmonious life in academic arena and outside. This attribute can germinate many positive outcomes in L2 education for teachers and students including their psych-emotional development, interpersonal communication skills’ formation, (inter)cultural sensitivity and awareness, ambiguity and diversity tolerance, socio-interactional competence growth, and many more.

### The Concept of Immediacy: Definitions and Typologies

Immediacy, in educational jargon, refers to the utilization of different communication cues and behaviors to reduce the existing psychological/physical distance among students and teachers (Christophel and Gorham, 1995). It is the use of verbal, non-verbal, and a mixture of other expressive strategies used by teachers and students to establish a positive rapport in the classroom and form a sense of liking that facilitates the ground for other academic achievements to emerge (Dickinson, 2017; Zheng, 2021). As for its typologies, research proposed different types of immediacy in education divided into verbal and non-verbal categories. Verbal immediacy, as put by Ballester (2013), refers to verbal messages that indicate a sense of empathy, reward, praise, willingness, openness, kindness, inclusiveness, and humor in classroom communication. On the other hand, non-verbal teacher immediacy cues aim to form physical and emotional proximity among students and the teacher to increase their attention, classroom engagement, and liking toward their teacher (Richmond and McCroskey, 2000). Basically, EFL teachers use non-verbal immediacy behaviors to create a sense of closeness, liking, and warmth and transfer such a feeling to students. Examples of non-verbal cues include *proxemics* (distance), *haptics* (touch), vocalics (paralanguage features- pitch, tone, stress, intonation, gesture, posture, etc.), *kinesics* (body movement/orientation), *oculesics* (eye contact), *classroom environment* (e.g., seating arrangements), and *chronemics* (time) (Stilwell, 2018).

### Theoretical Foundations of Immediacy

Different theories may directly and indirectly underlie teacher immediacy in education. However, Bowlby’s (1969) attachment theory (AT) and Maslow’s (1962) hierarchy of needs theory are the most relevant ones as per this article. AT, as a cornerstone of developmental psychology, explains the relational patterns among people. It maintains that one’s attachment to other individuals constitutes a behavior in the person that can become self-directed in the future. The theory has recently entered language education (Riley, 2011; Zheng, 2021) to highlight the role of emotional bonds among individuals in forming classroom rapport, practices, experiences, and involvement in activities (Zheng, 2021). Depending on the degree of emotion-sensitivity ad liking, EFL students establish emotional connections with their teachers and peers that can be either secure or insecure.

According to AT, students with affective attachment to their teacher are more relaxed, risk-takers, socialized, highly motivated, and engaged (Bergin and Bergin, 2009; Wang and Guan, 2020). In approachable atmospheres as such, teachers’ relationship and rapport with their pupils will be secure and valuable. Hence, the students feel free to express their ideas without flushing out. This sense of attachment fertilizes the ground for students’ growth and academic development.

**FIGURE 1:**
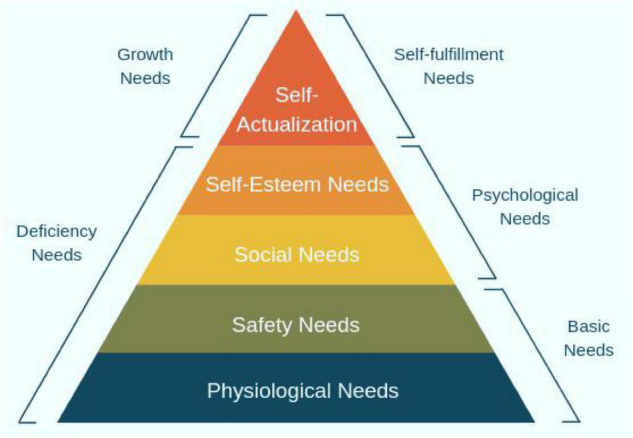
The hierarchy of needs theory (Maslow, 1962).

Another pertinent theory in this study is the hierarchy of needs theory (Maslow, 1962) that was a leap forward at its time (Figure 1). It argued that one’s basic human needs must be fulfilled so that he/she is able to show ultimate performance (Freitas and Leonard, 2011). Based on this pyramid of needs, as the basic and lower ranked needs are met, one’s motivation and enthusiasm to make more efforts to promote his/her ability increase as well (Maslow, 1962).

Unquestionably, all these layers of human needs are critical in life and education. Nevertheless, the psychological ones (belongingness and intimacy in communities) are considered more important in academia as they can compensate for the deficiencies in other layers and increase students’ motivation to learn/attempt. That is why, it is believed that effective teachers who make strong emotional ties with their students use various immediacy cues to show their care for the psychological needs of their students (Teven and Hanson, 2004). Additionally, it is worth mentioning that the social needs of the teachers and students in the pyramid justify the other two important variables in this review article (open-mindedness and SI).

### Social Intelligence: Conceptualizations and Models

As a significant type of intelligence tantamount to interpersonal intelligence proposed in Gardner’s (1983) multiple intelligence theory, SI is defined as a combination of self- and social-awareness, settled social beliefs and attitudes, and ability to cope with complicated social changes (Ganaie and Mudasir, 2015). It is one’s capacity to effectively interact with people and form human relations wisely (Thorndike, 1920). According to Marlowe (1986), SI is the capability to understand self and others’ feelings, thoughts, and behaviors in interpersonal communications and to act properly using that understanding. Despite these definitions, the literature in this domain has not yet complied on a universal and solid theoretical and operational definition for this type of intelligence (Grieve and Mahar, 2013). However, there are three models for SI that can help solve the problem and cast more light on this construct in L2 education.

The first one is the Salovey and Mayer’s (1990) model that considers SI as the capacity to perceive, comprehend, manage and utilize emotions to ease thinking. Trying to cover more components and aspects, Goleman (1998) proposed another model for SI and EQ and regarded SI as a construct shaped by a range of competencies and skills (self-regulation, self-awareness, relationship skills, and social awareness) that determine one’s performance. The last model is the Bar-On (1997, 2006) model that proposes five interrelated emotional and social competencies, skills, and facilitators (interpersonal skills, decision-making, self-expression, self-participation, and stress management) that affect one’s intelligent behavior.

### Correlates of Teacher Social Intelligence

Although the construct of SI has been overlooked by L2 scholars in comparison to other types of intelligence, in the past couple of years the concept has been empirically studied in relation to variables such as teacher reflectivity, effectiveness, intercultural sensitivity, critical thinking, cultural intelligence, and spiritual intelligence (see Genç and Boynukara, 2017; Nurfaidah, 2018). Despite these breakthroughs, the investigation of SI along with positive interpersonal communication skills (clarity, credibility, and immediacy), positive emotions, and personal attributes like open-mindedness has been drawn a veil over in the context of L2 education. As SI has to do with people interaction, many other correlates pertaining to social awareness and cultural diversity may also have a role in teachers’ SI formation and development.

### Final Remarks

In light of this mini review, it was contended that EFL classrooms as a representation of social contexts involve various interpersonal interactions as the syllabi these days are interactional in L2 education. To perform best in such contexts, EFL teachers must hone their skills to accept opposing views expressed by others and with the help of their students, establish a positive classroom climate wherein a sense of proximity is created among students and teachers. This sense of belongingness and immediacy paves the way for many other optimal academic outcomes for both students and instructors. One of such benefits concerns the development of teachers’ SI which is a necessary feature of effective L2 teachers. Based on these arguments, it can be stated that this article can be beneficial for EFL teachers, teacher educators, researchers, and materials developers.

EFL teachers may find this mini review helpful in that it can increase their awareness of social, interactional, and psych-emotional factors that influence their pedagogy. They can also use this study as a starting point for cultivating the variables of concern in their students using reflective practices. Furthermore, teacher educators may benefit from this study and design professional development courses for pre-service and in-service EFL teachers of different experience levels regarding the role of open-mindedness, interpersonal communications skills (e.g., immediacy), and SI in the process of L2 teaching and learning. Likewise, L2 researchers can use the ideas posed in this review and conduct similar studies focusing on cross-cultural disparities as per the three variables covered in this article. They can also add to the literature by running studies on the correlation of other types of intelligence and interpersonal communications skills *via* qualitative and mixed-methods designs. Another possible line of research can be offering developmental courses on EFL teachers’ open-mindedness, immediacy, and SI to see if they can be developed through instruction or not. Finally, materials developers in EFL can take this study and begin writing and incorporating materials and tasks that can cover and develop various types of intelligence and interpersonal communications skills in both teachers and students which has been regretfully ignored in many contexts.

## Author Contributions

The author confirms being the sole contributor of this work and has approved it for publication.

## Conflict of Interest

The author declares that the research was conducted in the absence of any commercial or financial relationships that could be construed as a potential conflict of interest.

## Publisher’s Note

All claims expressed in this article are solely those of the authors and do not necessarily represent those of their affiliated organizations, or those of the publisher, the editors and the reviewers. Any product that may be evaluated in this article, or claim that may be made by its manufacturer, is not guaranteed or endorsed by the publisher.
